# Combining Clinical and Genomic Covariates via Cov-TGDR

**Published:** 2007-10-15

**Authors:** Shuangge Ma, Jian Huang

**Affiliations:** 1 Department of Epidemiology and Public Health, Yale University, New Haven, CT, U.S.A; 2 Departments of Statistics and Actuarial Science, University of Iowa, Iowa City, IA, U.S.A

**Keywords:** classification, microarray, regularized estimation, survival analysis

## Abstract

Clinical covariates such as age, gender, tumor grade, and smoking history have been extensively used in prediction of disease occurrence and progression. On the other hand, genomic biomarkers selected from microarray measurements may provide an alternative, satisfactory way of disease prediction. Recent studies show that better prediction can be achieved by using both clinical and genomic biomarkers. However, due to different characteristics of clinical and genomic measurements, combining those covariates in disease prediction is very challenging. We propose a new regularization method, Covariate-Adjusted Threshold Gradient Directed Regularization (Cov-TGDR), for combining different type of covariates in disease prediction. The proposed approach is capable of simultaneous biomarker selection and predictive model building. It allows different degrees of regularization for different type of covariates. We consider biomedical studies with binary outcomes and right censored survival outcomes as examples. Logistic model and Cox model are assumed, respectively. Analysis of the Breast Cancer data and the Follicular lymphoma data show that the proposed approach can have better prediction performance than using clinical or genomic covariates alone.

## 1. Introduction

Tremendous effort has been devoted to discovering biomarkers that can be used in prediction of disease occurrence and progression. Clinical covariates—such as age, gender, blood pressure, tumor size and grade, and smoking and drinking history—have been extensively used and shown to have satisfactory predictive power (Gajdos et al. 1999; Negri et al. 2005). Clinical risk factors have sound biological implications and are usually easy to measure and of low dimensionality.

Recent developments in high throughput techniques, such as microarray, make it possible to measure human genomic features on a global scale. Biomedical studies with high dimensional gene expressions measured along with disease outcomes are becoming commonplace (Dave et al. 2004; Rosenwald et al. 2003; Alizadeh et al. 2000). Scientists have shown that using genomic biomarkers selected from microarray measurements may provide satisfactory prediction of disease status. See for example van’t Veer et al. (2002) and Shipp et al. (2002), among others. Using genomic measurements provides an alternative, satisfactory way of disease prediction beyond clinical covariates.

Clinical and genomic covariates may correspond to different aspects of causation of diseases. Consider the occurrence of lung cancer as an example. Studies have shown that smoking, which is a clinical covariate, is the best predictor of lung cancer occurrence. How ever, genetic defection has also been shown to contribute to occurrence of lung cancer. By combining smoking history with genomic measurements, prediction with better sensitivity and specificity (than using smoking or genetic defection alone) can be achieved. Such an improvement has been observed with other diseases (Rosenwald et al. 2002; Pittman et al. 2004). It is thus of great interest to develop statistical methodologies that can effectively combine low dimensional clinical and high dimensional genomic measurements in disease prediction.

In Fernandez-Teijeiro et al. (2004), a small number of genes are first selected and then combined with clinical covariates in predictive model building. Such an approach, although very easy to implement, ignores clinical covariates in gene selection and may lead to sub optimal results. In Ghosh and Chinnaiyan (2005), adjusting for clinical covariates in detecting differential genes is investigated in the linear regression and FDR framework. In that study, the goal is to detect differentially expressed genes, and predictive model building is not considered. A sufficient dimension reduction approach is proposed by Li (2006) in the framework of survival analysis, where two lymphoma survival datasets are analyzed. The sufficient dimension reduction method uses linear combinations of all covariates, which makes it hard to interpret individual covariate effects. In a breast cancer study with a binary response representing the disease status, Sun et al. (2007) proposes the iterative 1-RELIEF approach. It is not clear how to extend that approach to studies with other clinical outcomes such as survival.

In this article, we propose a new regularized method, Cov-TGDR (Covariate-Adjusted Threshold Gradient Directed Regularization), for combining different type of covariates in disease prediction. The proposed approach is capable of simultaneous biomarker selection and predictive model building. It has great flexibility by allowing different degrees of regularization for different type of covariates. The rationale is that clinical and genomic covariates are not directly comparable. Different regularization should thus be considered. Similar arguments have been made in Li (2006) and Sun et al. (2007). In our study, we only consider two type of covariates, namely clinical and genomic. In principle, the proposed Cov-TGDR can be used when more than two type of covariates are present.

In Section 2, we first present the data and models that we consider. We use logistic regression for binary classification and Cox model for right censored survival analysis as examples. The proposed Cov-TGDR is described in Section 3. Tuning parameter selection and prediction evaluation are also discussed. We present analysis of the Breast Cancer data (which has a binary outcome) in Section 4 and analysis of the Follicular lymphoma data (which has a right censored survival outcome) in Section 5, respectively. The article concludes with discussions in Section 6.

## 2. Data and Model

Let *Y* be the clinical outcome of interest. Let *Z* = (*W*, *X*) be the length *d* vector of covariates. Specifically, let *W* be the length *d*_1_ vector consisting of clinical covariates; and let *X* be the length *d*_2_ vector of gene expressions, where *d*_1_ + *d*_2_ = *d*. In a typical biomedical study, *d*_1_ ∼ 10 while *d*_2_ ∼ 10^3–4^. For simplicity of notations, we assume there are only two different sets of covariates. The proposed approach can be easily extended to multiple sets of covariates.

Suppose that *Y* is associated with *Z* through the model *Y* ∼ *φ*(*β′Z*) with known regression function *φ* and unknown regression coeffcient *β*. We are particularly interested in classification and survival analysis problems where both clinical and genomic covariates are measured along with disease outcomes due to their extensive applications.

### 2.1 Binary classification

For classification problems, *Y* is the categorical variable denoting the disease status. For simplicity of notations, we focus on binary classification only. Suppose that *Y* = 1 representsthe presence and *Y* = 0 indicates the absence of disease. We assume the commonly used logistic regression model, where the logit of the conditional probability is

$$\text{logit}(P(Y=1|Z)=\alpha +\beta^{\prime }Z$$

Here *β* is the length *d* vector of regression coeffcient and α is the intercept. Based on a random sample of *n* iid observations (*Y_i_*, *Z_i_*)*, i* = 1, ..., *n*, the maximum likelihood estimator is defined as (*α̂*, *β̂*) = *argmax_α, β_* *R_n_*(*α*, *β*), where

$$\begin{array}{l} R_{n}(\alpha ,\beta )=\sum_{i=1}^{n}Y_{i}\text{log}\left(\frac{\text{exp}(\alpha +\beta^{\prime }Z_{i})}{1+\text{exp}(\alpha +\beta^{\prime }Z_{i})}\right)\\ \;+(1-Y_{i})\text{log}\left(\frac{1}{1+\text{exp}\left(\alpha +\beta^{\prime }Z_{i}\right)}\right) \end{array}$$

Since *α* is usually of secondary interest, we simply write *R_n_*(*α*, *β*) as *R_n_*(*β*).

### 2.2 Cox survival analysis

For right censored survival data, *Y* = (*T*, Δ), where *T* = *min*(*U*, *V*) and Δ = *I* (*U* ≤ *V*). Here *U* and *V* denote the event and censoring times, respectively. The most widely used model for censored survival data is the Cox model (Cox, 1972) which assumes that the conditional hazard function

$$\lambda (u|Z)=\lambda_{0}(u)\text{exp}(\beta^{\prime }Z)$$

where λ_0_ is the unknown baseline hazard function and *β* is the unknown regression coeffcient. Based on a random sample of *n* iid observations (*Y_i_*, *Z_i_*), *i* = 1, ..., *n*, the partial likelihood estimator is defined as the value *β̂* that maximizes

Rn(β)=∏i=1n{exp(β′Zi)∑j∈riexp(β′Zi)}δi

where *r_i_* = {*j: T_j_* ≥ *T_i_*} is the risk set at time *T_i_*.

For both logistic classification and Cox survival analysis, *β* can be estimated by maximizing the continuously differentiable likelihood or partial likelihood functions, which depend on *β* only. The proposed Cov-TGDR is generally applicable if other parametric or semiparametric models are assumed, provided that smooth objective functions are available.

## 3. Cov-TGDR

### 3.1 Algorithm

The proposed Cov-TGDR is a gradient searching approach. We refer to Friedman and Popescu (2004) for background and general discussions on such an approach. Let Δ*ν* be a small positive increment. In the implementation of our approach, we choose Δ*ν* = 1 × 10^−3^. Denote *ν_k_* = *k* × Δ*ν* as the index for the point along the parameter path after *k* steps. Let *β* (*ν_k_*) denote the parameter estimate corresponding to *ν_k_*. Denote 0 ≤ τ_1_,τ_2_ ≤ 1 as the threshold values for clinical and genomic covariates, respectively. The proposed Cov-TGDR consists of the following iterative steps:

1. Initialize *β* (0) = 0 and *ν*_0_ = 0.
2. With current estimate *β*, compute the negative gradient *g*(*ν*) = −*∂R_n_*(*β*)*/∂β*. Denote the *jth* component of *g*(*ν*) as *g_j_*(*ν*). If max*j* {| *g_j_*(*ν*)|} = 0, stop the iteration.
3. Compute the length-*d* threshold vector *f* (*ν*), where
  $$\begin{array}{l} f_{j}(v)=\;I\{\left|g_{j}(v)\right|\geq \tau_{1}\times \underset{1\leq l\leq d_{1}}{\text{max}}\left|g_{l}(v)\right|\},\\ \;\;\;\;\;\;\;\;\;\;\;\;\;\;\;\;\;\;\;\;\;\;\;\;\;\;\;\;\;\;\;\;\;\;\;\;\;\;\;\;\;\;\;\;\;\;\;\;\;\;\;\;\;\;\;\;\text{for}\;1\leq j\leq d_{1}\\ f_{j}(v)=\;I\{\left|g_{j}(v)\right|\geq \tau_{2}\times \underset{l>d_{1}}{\text{max}}\;\left|g_{l}(v)\right|\},\\ \;\;\;\;\;\;\;\;\;\;\;\;\;\;\;\;\;\;\;\;\;\;\;\;\;\;\;\;\;\;\;\;\;\;\;\;\;\;\;\;\;\;\;\;\;\;\;\;\;\;\;\;\;\;\;\;\;\;\;\;\text{for}\;j>d_{1} \end{array}$$
4. Update *β* (*ν_k_* + Δ*ν*) = *β* (*ν_k_*) − Δ*ν* × *g*(*ν_k_*) × *f* (*ν_k_*) and update *ν_k_* by *ν_k_* + Δ*ν*, where the product of *f* and *g* is component-wise.
5. Steps 2–4 are repeated *k* times. The number of iterations *k* is determined by cross validation.

The Cov-TGDR uses a thresholding and variable selection scheme quite different from the TGDR in Friedman and Popescu (2004). Particularly in Step 3, thresholding is carried out for different sets of covariates separately. The rationale is that different type of covariates are not directly comparable—one unit increase in gene expressions may have quite different implications from one unit increase in clinical covariates. In addition, genomic covariates usually have a much higher dimensionality than clinical covariates. Variable selection is much more important for genomic covariates than for clinical covariates, which demands a higher degree of regularization for genomic covariates. A fair approach should consider thresholding comparisons within each type of covariates separately, as in Step 3.

Loosely speaking, the Cov-TGDR carries out TGDR for each type of covariates separately. The properties of *β* are determined jointly by *k* and (*τ*_1_,*τ*_2_). When (*τ*_1_,*τ*_2_) = (0, 0), the Cov-TGDR does not carry out biomarker selection and generates estimates similar to the ridge regression. When (*τ*_1_ = 0,*τ*_2_ > 0), the Cov-TGDR carries out variable selection with gene expressions, while adjusting for clinical covariates without any variable selection. When (*τ*_1_ > 0,*τ*_2_ > 0), variable selections are carried out for both clinical and genomic covariates. By allowing *τ*_1_ ≠ *τ*_2_, the proposed Cov-TGDR is more flexible than the TGDR.

In addition, it takes into account clinical covariates when estimating and selecting variables with gene expressions. It is thus more reasonable than the naive approach, where TGDR estimations are carried out separately for clinical and genomic covariates.

### 3.2 Tuning parameter selection

We select the tuning parameters *k* and (*τ*_1_,*τ*_2_), which jointly determine the characteristics of the estimator, using the following two-step approach. First, we choose the tuning parameter *k* for any fixed (*τ*_1_,*τ*_2_) using the *V*-fold cross validation (Wahba, 1990) as follows. Partition the data randomly into *V* non-overlapping subsets of equal sizes. Choose *k* to maximize the cross-validated objective function

(1) $$CV(k)=\sum_{\upsilon =1}^{V}\left[R_{n}(\beta^{\left(-\upsilon \right)})-R_{n}^{\left(-\upsilon \right)}(\beta^{\left(-\upsilon \right)})\right]$$

where *β*^(−^*^υ^*^)^ is the Cov-TGDR estimate of *β* based on data without the *υ*th subset for a fixed *k* and *R_n_*^(−^*^υ^*^)^ is the objective function *R_n_* evaluated without the *υ*th subset. Considering the relatively small sample sizes, we set *V* = 5 in our study.

After cross validation over *k*, model features for different (*τ*_1_,*τ*_2_) can be obtained. We choose parsimonious models with relatively large CV scores. A similar approach has been adopted in Ma and Huang (2005) and references therein.

### 3.3 Evaluation

Prediction evaluation can be based on the following Leave-One-Out (LOO) approach. For *i* = 1, ..., *n*,

1. Remove the *i*th subject.
2. For the reduced dateset with size *n* − 1, carry out the V-fold cross validation and Cov-TGDR estimation. Denote this estimate as *β̂*^(−^*^i^*^)^.
3. Compute the prediction score *β̂*^(−^*^i^*^)^′ *Z_i_* for the removed subject.

A prediction index can then be computed. For binary classification, class probabilities can be computed from the prediction scores and the logistic model. We use probability 0.5 as the cutoff and predict disease status for each subject. The prediction index can be chosen as the prediction error. For censored survival data, we dichotomize the prediction scores at their median and create two hypothetical risk groups. We then compare the survival functions of the two risk groups. The logrank statistic, which has a Chi-squared distribution with degree of freedom one, is taken as the prediction index.

## 4. Breast Cancer Study

Breast cancer is the second leading cause of deaths from cancer among women in the United States. Despite major progresses in breast cancer treatment, the ability to predict the metastatic behavior of tumor remains limited. The Breast Cancer study was first reported in van’t Veer et al. (2002). 97 lymph node-negative breast cancer patients 55 years old or younger participated in this study. Among them, 46 developed distant metastases within 5 years (metastatic outcome coded as 1) and 51 remained metastases free for at least 5 years (metastatic outcome coded as 0).

Clinical covariates collected include age, tumor size, histological grade, angioinvasion, lymphocytic infiltration, estrogen receptor (ER), and progesterone receptor (PR) status. Expression levels for 24481 gene probes were collected. We refer to van’t Veer et al. (2002) for more details on experimental setup. The goal of this study is to build a statistical model that can accurately predict the risk of distant recurrence of breast cancer in a five-year post-surgery period. The dataset is publicly available at *http://www.rii.com/publications/2002/vantveer.html.*

We first pre-process gene expression data as follows:

1. Remove genes with more than 30% missing measurements.
2. Fill in missing gene expression measurements with median values across samples.
3. Normalize gene expressions to have zero means and unit variances.
4. Compute the simple correlation coefficients of gene expressions with the binary outcome.
5. Select the 500 genes with the largest absolute values of correlation coefficients.

It is reasonable to expect that the number of “interesting” genes is much less than 500 (see Ma and Huang, 2005 and references therein); In addition, including many “noisy” genes in the biomarker selection and model building may lead to less satisfactory results. We thus conduct gene screening prior to the analysis and select only the top 500 genes (Sun et al. 2007; Ma, 2006).

**Figure 1.**
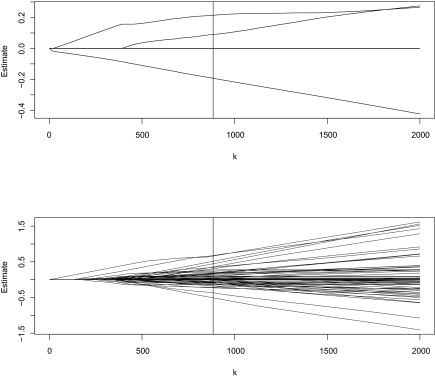
Breast cancer data. Parameter paths as a function of *k* for (*τ*_1_,*τ*_2_) = c(1.0, 0.9). Upper panel: clinical covariates; Lower panel: genomic covariates.

The proposed Cov-TGDR is used to analyze the Breast Cancer data. The 5-fold cross validation selects *k* = 884 and (*τ*_1_,*τ*_2_) = (1.0, 0.9) as the optimal tunings. We show the parameter paths as a function of *k* for (*τ*_1_,*τ*_2_) = (1.0, 0.9) in Figure 1. The vertical lines correspond to *k* = 884. Since both threshold values are large, the parameter paths look like Lasso paths – they start with all estimates equal to zero; the estimates remain sparse for moderate to large *k* ; and the estimates eventually become dense as *k* → ∞. Similar phenomenon has been observed in Friedman and Popescu (2004) and Ma and Huang (2005).

With the optimal tuning, the final predictive model includes 3 (out of 7) clinical covariates and 51 (out of 500) genomic biomarkers. We list covariates with nonzero estimated coeffcients in Table 1. The three important clinical covariates are age, tumor diameter, and tumor grade, which have long been used as risk factors for predicting breast cancer. Especially, increase of tumor size or grade indicates worsening or proliferation of tumor, which leads to higher likelihood of cancer occurrence. Moreover, our analysis shows that after ad justing for other risk factors, older people are less likely to develop breast cancer. We note that this conclusion cannot be extended to the general population, since the current study only included patients 55 years old or younger. We also provide systematic names and corresponding estimates for identified genes. Gene names and corresponding annotations can be found from the data website and *http://www.ncbi.nlm.nih.gov/*. Many of the identified genes have been shown to be associated with breast cancer occurrence in independent studies. We refer to van’t Veer et al. (2002) for detailed discussions of gene functions.

For comparison, we also consider three closely related alternatives: (1) Clinical-simple: only clinical covariates are used in the analysis. Since the number of clinical covariates is less than the sample size, logistic model without any regularization can be fitted; (2) Clinical-TGDR: only clinical covariates are used in the analysis, and we use TGDR for regularization. With the TGDR, tuning parameters include the number of iterations *k* and threshold *τ*, (3) Gene-TGDR: only gene expressions are used. TGDR is employed for gene selection and regularized estimation. For alternative approaches (2) and (3), we also use the 5-fold cross validation to select optimal tunings. Prediction evaluation is carried out for all four approaches using the LOO described in Section 3.3. In our estimation, we conduct gene screening prior to the analysis. In the evaluation, for each reduced dataset with size *n* − 1, we also carry out gene screening and select (possibly different sets of) 500 top genes. Since gene screening is included in the LOO, the prediction evaluation has no selection bias.

Estimation and prediction results are summarized in Table 2. We can see that using clinical covariates alone without any regularization results in less satisfactory prediction. With clinical covariates, using TGDR for regularization can reduce model size and increase prediction power. Using gene expressions alone can lead to improved prediction, with the larger model as payoff. Prediction can be further improved by using both clinical and genomic covariates, although the resulted model is larger than all alternatives.

## 5. Follicular Lymphoma Study

Follicular lymphoma is the second most common form of non-Hodgkin’s lymphoma, accounting for about 22 percent of all cases. A study was conducted to determine whether the survival risks of patients with follicular lymphoma can be predicted by the gene-expression profiles of the tumors and standard clinical risk factors at diagnosis (Dave et al. 2004). Detailed experiment setup and raw data can be accessed at *http://llmpp.nih.gov/FL/*.

Fresh-frozen tumor-biopsy specimens and clinical data from 191 untreated patients who had received a diagnosis of follicular lymphoma between 1974 and 2001 were obtained. The median age at diagnosis was 51 years (range: 23 to 81), and the median follow up time was 6.6 years (range: less than 1.0 to 28.2). The median follow up time among patients alive at last follow up was 8.1 years. Eight records with missing survival information are excluded from the analysis.

Clinical covariates measured include extra nodal site, age, normalized LDH, performance status, stage and IPI.1 (IPI value equal to 2 or 3), and IPI.2 (IPI value equal to 4 or 5). We remove subjects with missing clinical covariate measurements. 156 subjects are included in the Cov-TGDR analysis. Affymetrix U133A and U133B microarray genechips were used to measure gene expression levels. A log2 transformation was first applied to the Affymetrix measurements. We filter the 44928 gene measurements with the following criteria: (1) the max expression value of each gene across 156 samples must be greater than the median max expressions; and (2) the max–min expressions should be greater than their median. 6506 out 44928 genes pass the above unsupervised screening. We further compute the correlation coeffcients of the uncensored survival times with gene expressions. The 500 genes with the largest absolute values of the correlation coeffcients are selected.

We apply the proposed Cov-TGDR. Parameter paths similar to those shown in Figure 1 can be obtained and are omitted here. With the Cov- TGDR, 6 (out of 7) clinical covariates and 23 (out of 500) genomic covariates are selected in the final model. We provide covariates with nonzero estimated coeffcients in Table 3. All measured clinical covariates have importance influences on survival risks. For the IPI measurement, only IPI.1 (IPI value equal to 2 or 3) is important. Increase of any clinical covariates will lead to increased survival risk. For gene expressions, with the Affymetrix feature IDs provided in Table 3, gene names and corresponding biological functions can be found from *http://llmpp.nih.gov/FL/*. Many identified genes have been confirmed by independent studies to be associated with survival risks in lymphoma patients. We omit such discussions here.

For the Cov-TGDR and alternative approaches, model estimation and prediction results are summarized in Table 4. As discussed in Section 3.3, we use the logrank statistic as the prediction index for censored survival data, with larger logrank statistic indicating more powerful prediction. We can see from Table 4 that using clinical covariates alone can lead to quite satisfactory predictions, with logrank statistics 17.9 and 18.1 and corresponding p-values <0.001. Using gene expression data alone, 31 genes are selected with the TGDR. The prediction logrank statistic is 4.0, corresponding to p-value 0.045. Prediction can be improved by using both clinical and genomic covariates (logrank statistic 23.9, p-value < 0.001).

## 6. Discussions

Given that clinical and genomic factors may contribute to different aspects of disease occurrence, it is important to use both for predicting disease status. We propose the Cov-TGDR method, which can achieve improved prediction by effectively combining those two type of covariates. The proposed Cov-TGDR is more flexible than the TGDR by allowing different degrees of regularization for different type of covariates. Especially, our numerical studies suggest that Cov-TGDR usually has *τ*_1_ ≤ *τ*_2_, i.e. less regularization is employed for clinical covariates. Another valuable feature of the Cov-TGDR is that the computational cost is small. For the Breast Cancer data, cross validation and estimation combined take less than two minutes. Compared to existing approaches, the Cov-TGDR generates smaller models than the sufficient dimension reduction method of Li (2006). The Cov- TGDR estimation results are thus easier to interpret. Compared to the 1-RELIEF approach of Sun et al. (2007), the proposed Cov-TGDR depends less on the form of the objective function. It can be easily adapted to studies with other type of outcomes and models.

Like in Li (2006) and Sun et al. (2007), the proposed Cov-TGDR is built on an existing regularization method (i.e. TGDR). However, they differ significantly in terms of thresholding and variable selection scheme. The two presented studies and other examples (not presented here) show that improved prediction can be achieved with the proposed Cov-TGDR. We note that the improvement may not be as dramatic as one may expect. However, considering the difficulties with predicting status of complicated diseases such as cancer, even very small improvement may have extremely important clinical implications, as has been observed in previous studies (Li, 2006).

One drawback of our study is that no theoretical justification is available for the proposed Cov- TGDR. The proposed estimate is a non-linear function of the observations, which makes it difficult to establish its theoretical properties, such as consistency in terms of variable selection under reasonable conditions. Our limited numerical study establishes the Cov-TGDR’s satisfactory empirical performance. More studies are needed to understand its theoretical properties.

## Figures and Tables

**Table 1.** Breast Cancer Data: Cov-TGDR estimation. Variable: variable name (clinical) or systematic name (genomic).

**Variable**	**Estimate**	**Variable**	**Estimate**
Clinical covariates			
age	−0.193		
diameter	0.090		
grade	0.214		

Genomic covariates			
AB033032	0.007	AJ011306	−0.214
Contig5816_RC	0.169	NM_013438	0.045
Contig35148_RC	−0.368	NM_004994	0.142
Contig46909_RC	−0.230	AL080059	0.660
Contig23356_RC	0.097	Contig42563_RC	0.087
Contig35229_RC	−0.134	NM_006544	0.159
Contig28433_RC	−0.014	NM_005850	0.005
NM_003366	−0.068	Contig64861_RC	0.194
NM_020120	0.038	AF055033	0.514
NM_020123	0.343	NM_016017	0.037
NM_020132	0.012	Contig47544_RC	0.674
U72507	−0.089	Contig48697_RC	0.029
Contig6238_RC	−0.116	NM_016361	−0.174
AF052087	−0.083	NM_016448	0.029
NM_005007	−0.082	Contig412_RC	−0.510
AB018337	0.270	NM_016564	0.445
AB040969	0.010	NM_018089	0.178
NM_012341	−0.033	D13540	0.089
Contig47042	0.189	U79298	−0.177
Contig38438_RC	−0.096	NM_000127	0.234
X67055	−0.005	NM_019018	−0.074
NM_003862	−0.138	NM_000207	−0.049
NM_003882	−0.083	AL050227	−0.010
AF131819	0.356	Contig22253_RC	−0.012
NM_014003	0.120	NM_000801	0.059
NM_005393	0.304		

**Table 2.** Analysis of Breast Cancer Data. # clinical: number of clinical variables. # gene: number of gene expressions. Tuning: optimal tuning parameters. Error: prediction error.

**Method**	**# clinical**	**# gene**	**Tuning**	**Error**
Clinical-simple	7	–	–	0.371
Clinical-TGDR	5	–	*τ* = 0.8	0.289
Gene-TGDR	–	50	*τ* = 0.9	0.267
Cov-TGDR	3	51	(*τ*_1_, *τ*_2_) = (1.0, 0.9)	0.227

**Table 3.** Follicular Lymphoma Data: Cov-TGDR estimation. Variable: variable name (clinical) or Affymetrix Feature ID (genomic).

**Variable**	**Estimate**	**Variable**	**Estimate**
Clinical covariates			
nodal	0.123	pstat	0.194
age	0.450	stage	0.309
ldh	0.469	IPI.2	0.514

Genomic covariates			
223710_at	−0.108	240593_x_a	0.006
225981_at	0.222	201739_at	−0.020
226587_at	0.004	202783_at	−0.040
230280_at	0.066	203612_at	0.040
232204_at	−0.050	212713_at	−0.028
232883_at	0.066	215536_at	−0.126
234062_at	−0.036	208470_s_a	0.214
235058_at	−0.004	216950_s_a	0.012
239565_at	0.016	217893_s_a	−0.110
224280_s_a	−0.202	219360_s_a	0.056
230938_x_a	0.054	220235_s_a	−0.090
234792_x_a	0.054		

**Table 4.** Analysis of Follicular lymphoma Data. # clinical: number of clinical variables. # gene: number of gene expressions. Tuning: optimal tuning parameters. Logrank: logrank statistics.

**Method**	**# clinical**	**# gene**	**Tuning**	**Logrank**
Clinical-simple	7	–	–	17.9
Clinical-TGDR	6	–	*τ* = 0.1	18.1
Gene-TGDR	–	31	*τ* = 1.0	4.0
Cov-TGDR	6	23	(*τ*_1_, *τ*_2_) = (0.1, 1.0)	23.9
